# COMPASSIONATE RELEASE OF DYING INMATES: A STEP TOWARD SOCIAL JUSTICE IN THE CRIMINAL JUSTICE SYSTEM

**DOI:** 10.1093/geroni/igad104.1607

**Published:** 2023-12-21

**Authors:** Gabriel Lutz, Yulin Yang, Raya Kheirbek

**Affiliations:** University of Maryland School of Medicine, Baltimore, Maryland, United States; University of California, San Francisco, San Francsico, California, United States; University of Maryland School of Medicine, Baltimore, Maryland, United States

## Abstract

Mr. John is a 60-year-old incarcerated male with advanced stage IV lung cancer that had metastasized. He was admitted to the hospital with significant pain and failure to thrive. Several lines of chemotherapy failed him, and he desired to be at home with his family as he approached the end of life. Unfortunately, he was unable to leave the hospital except to be back to prison. He was continually shackled to bed with 2 police officers in his room. The case highlights the need to advocate for compassionate release, that allows for the early release of terminally ill inmates who are not a danger to society, and offers tools and steps to help release hospitalized inmates to the care of their families. To obtain compassionate release, the first step is to identify eligible inmates with a terminal illness and a life expectancy of less than six months, which must be confirmed by a medical evaluation. The second step involves filing a petition for compassionate release with the appropriate authority, including medical records, a statement from the treating physician, and a plan for the inmate’s release and care in the community. The third step entails advocating for the inmate’s release, which may involve working with advocacy groups, contacting elected officials, and raising public awareness. By granting compassionate release to dying inmates, we can provide them with the opportunity to die with dignity and spend their final moments with their loved ones, which is a fundamental aspect of social justice.

